# Supplementary material for: Controlling wave fronts with tunable disordered non-Hermitian multilayers
Source: Sci Rep. 2021 Feb 26;11:4790. doi: 10.1038/s41598-021-84271-0 (PMC7910583; doi:10.1038/s41598-021-84271-0)
Supplement: Supplementary file 1 — Supplementary Information. [file 41598_2021_84271_MOESM1_ESM.pdf]

Supplementary Information for
Controlling wave fronts with tunable disordered non-Hermitian multilayers

Denis V. Novitsky[1], Dmitry Lyakhov[2], Dominik Michels[2], Dmitrii Redka[3], Alexander A. Pavlov[4], and Alexander S. Shalin[4,5]

[1]*B. I. Stepanov Institute of Physics, National Academy of Sciences of Belarus, Nezavisimosti Avenue 68, 220072 Minsk, Belarus*

[2]*Visual Computing Center, King Abdullah University of Science and Technology, Thuwal 23955-6900, Kingdom of Saudi Arabia*

[3] *Saint Petersburg, Electrotechnical University LETI (ETU), Prof. Popova Street 5, 197376 St. Petersburg, Russia*

[4] *Institute of Nanotechnology of Microelectronics of the Russian Academy of Sciences, Leninsky Prospekt 32A, 119991 Moscow, Russia*

[5]*Kotelnikov Institute of Radio Engineering and Electronics of the Russian Academy of Sciences (Ulyanovsk branch), Goncharova Str. 48, 432000 Ulyanovsk, Russia*

We have seen that changing the disorder strength allows to control propagation time of the wave front through the loss-gain multilayer. Another approach to such control is to use two waves in the counter-propagating fashion: the propagation time of a wave depends on the intensity of another one [1]. Here we study the combination of both approaches considering interaction of wave fronts in the presence of disorder.

We start with the case of two identical non-adiabatic fronts with one of them being "forward-propagating" and another being "backward-propagating". The parameters of calculation are the same as in corresponding section of the main text, the amplitude of the waves being $\Omega_0 = 10\gamma_2$. The results of calculation for different r are shown in Fig. S1. One can see that the time needed for the wave front to traverse the system and to establish the steady-state response becomes shorter in comparison to the single-front case (see Fig. 2 of the main text). This is in full accordance with the previous report [1]. On the other hand, at first, increase of disorder results in shortening propagation time (compare the cases of $r = 0$ and $r = 0.5$ in Fig. S1). Then, it grows again and at $r = 1$ is practically the same as at $r = 0$, in agreement with the regularities discussed for single fronts.

The similar features occur for the adiabatic fronts as shown in Fig. S2 for the same parameters as in the main text. The interacting kinks seen in panels (a) and (b) pass through the medium for the shorter time than the single kink. The only peculiarity is the low pedestal preceding the kink itself. This can be attributed to partial reflection of the counter-propagating wave. For larger disorder strengths ($r \gtrsim 0.5$), formation of the kinks is suppressed as well as for the single wave front (compare with Fig. 5 in the main text).

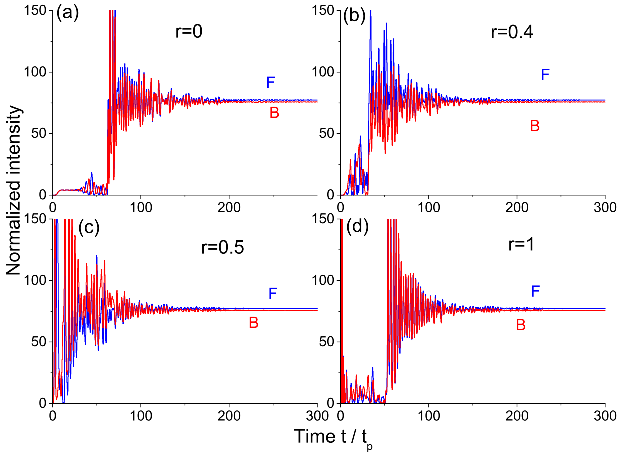

Figure S1. Intensity profiles for the forward (F) and backward (B) transmitted waves in the case of incident non-adiabatic fronts. Different panels show the results for different disorder strengths r.

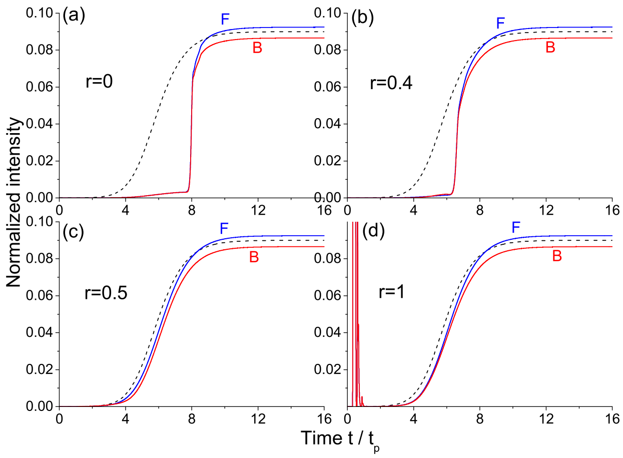

Figure S2. Intensity profiles for the forward (F) and backward (B) transmitted waves in the case of incident adiabatic fronts. Different panels show the results for different disorder strengths r.

[1] D. V. Novitsky, Optical kinks and kink-kink and kink-pulse interactions in resonant two-level media, Phys. Rev. A **95**, 053846 (2017).
